# Comments on "Investigations of the effects of upper extremity home exercises on grip strength, range of motion, activity performance, and functionality in individuals with systemic sclerosis: a randomized controlled trial"

**DOI:** 10.1590/1806-9282.20240153

**Published:** 2024-07-19

**Authors:** Pooja Sunil Patil, Amit Kumar, Shashi Prakash Sharma, Mahima Guleria

**Affiliations:** 1 Lovely Professional University, School of Allied Medical Sciences – Phagwara, India.

Dear Editor,

First and foremost, we extend our sincerest gratitude to the authors for their remarkable ability to articulate their thoughts with utmost clarity and conciseness on the published article entitled "Investigations of the effects of upper extremity home exercises on grip strength, range of motion, activity performance, and functionality in individuals with systemic sclerosis: a randomized controlled trial"^1^. This study has discovered that the implementation of upper extremity home exercises results in an augmentation of grip strength, range of motion, activity performance, and overall functionality among patients diagnosed with systemic sclerosis. We aim to draw attention to several methodological and statistical concerns pertaining to the study, with the purpose of enhancing the utilization of the study findings among healthcare practitioners who handle systemic sclerosis, ultimately leading to improvements in prognosis.

First, as indicated by the title and objective of the abstract, it is not evident that the study was conducted to determine the comparative effects of home exercises versus patients' education on patients with systemic sclerosis (Ssc). However, in the hypothesis section, the authors have discussed a comparison that may cause confusion among the readers. In the participants section, the authors have utilized the 2013 ACR/EULAR criteria for participant selection, but they have not clearly mentioned the scoring system. According to this criterion, a score of ≥9 is classified as definite Ssc^2^. The authors should have carefully specified the criteria to prevent negligence in future studies. Second, in the protocols section, specifically the upper extremity home exercises sub-section, the intervention group comprised patients performing home exercises. However, in the outcome measures section, grip strength and active/passive range of motion were measured using handheld dynamometer and goniometer, respectively. This may be perplexing for readers, as the authors have not mentioned the procedure for measuring these outcomes throughout the entire article. To our understanding, patients either visited the clinical setting or therapists visited the patients' homes for data collection. This crucial information is missing in the article.

Third, in the statistical analysis section, authors have not mentioned clearly about the statistical tests. According to normality, if data follow normal distribution, they should be interpreted in mean and standard deviation with parametric tests. If data do not follow normal distribution, they should be interpreted in median and interquartile range with non-parametric tests^3^. But in this study, authors have mentioned both types of tests (parametric and non-parametric), which may misinterpret the results. Intention-to-treat analysis could have been used by the authors as nine patients lost to follow-up^4^. The results would be incomplete without finding effect size and power analysis. Authors should have focused on this as this is the randomized controlled trial. From the aforementioned valid discussion, we advise the readers to proceed with caution in interpreting the results.
